# Supplementary material for: A Combination Adjuvant for the Induction of Potent Antiviral Immune Responses for a Recombinant SARS-CoV-2 Protein Vaccine
Source: Front Immunol. 2021 Sep 16;12:729189. doi: 10.3389/fimmu.2021.729189 (PMC8481386; doi:10.3389/fimmu.2021.729189)

Supplementary Material

**Supplemental Figure S1:** Antigen recall response assessed in lymphocytes from spleen and cLN isolated from mice immunized IM with 10 μg SARS-CoV-2 RBD alone, or with 50% Addavax in a volume of 50 μL according to a prime/boost/boost schedule (at a 4 wk interval). Cells were stimulated *ex vivo* with 5 μg of recombinant RBD for 72h, and levels of secreted IL-5 were measured in the supernatant relative to unstimulated cells by multiplex immunoassay. (*n*=5/grp; *p<0.05, **p<0.01 by Mann-Whitney U test)

**
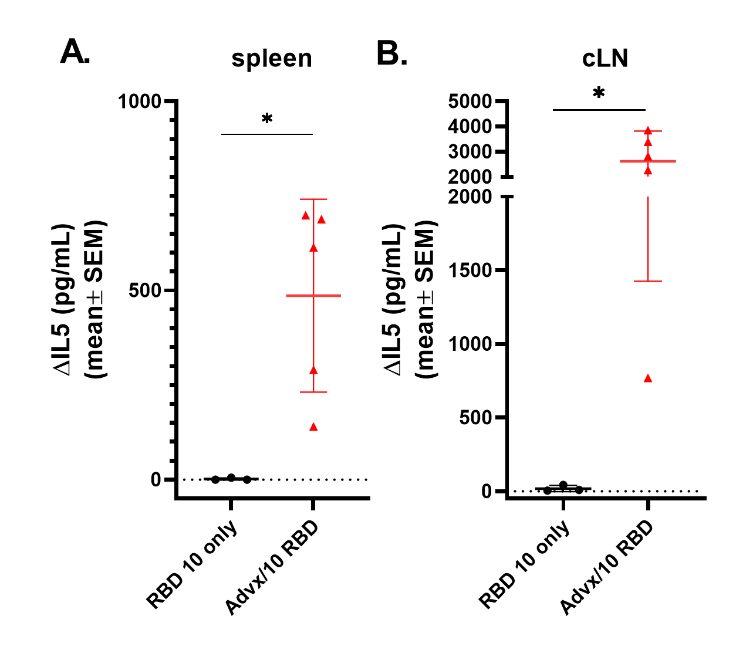
**

**Supplemental Figure S2.** Mucosal response assessed in immunized mice by measuring S1-specific IgA in bronchial alveolar lavage after prime/boost/boost immunizations (10 weeks post-initial immunization) as measured by ELISA. Absorbance values at 405 nm are shown after development with an alkaline-phosphatase conjugated secondary antibody with a pNPP substrate. (*p<0.05 by Mann-Whitney U test)


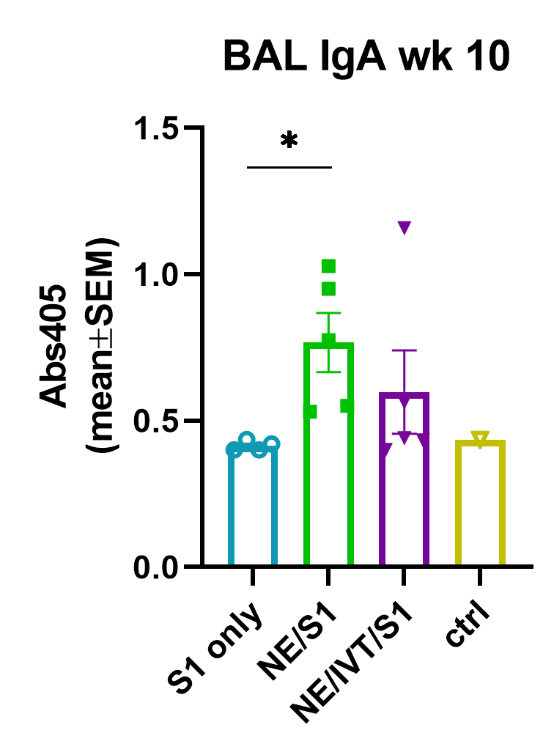


**Supplemental Figure S3.** **Serum S1-specific IgM and IgA titers induced by immunization with NE and NE/IVT.** (A) Serum S1-specific IgM measured in mice immunized IN with 15 μg S1 alone, or with 20% NE, or 20% NE/0.5 μg IVT DI measured 2 weeks after the first immunization. (B) Serum S1-specific IgA was measured 8 wks post-initial immunization (prime/boost), and (C) 10 wks post-initial immunization (prime/boost/boost). (*p<0.05, **p<0.01 by Mann-Whitney U test). Titers are shown as mean±SEM.


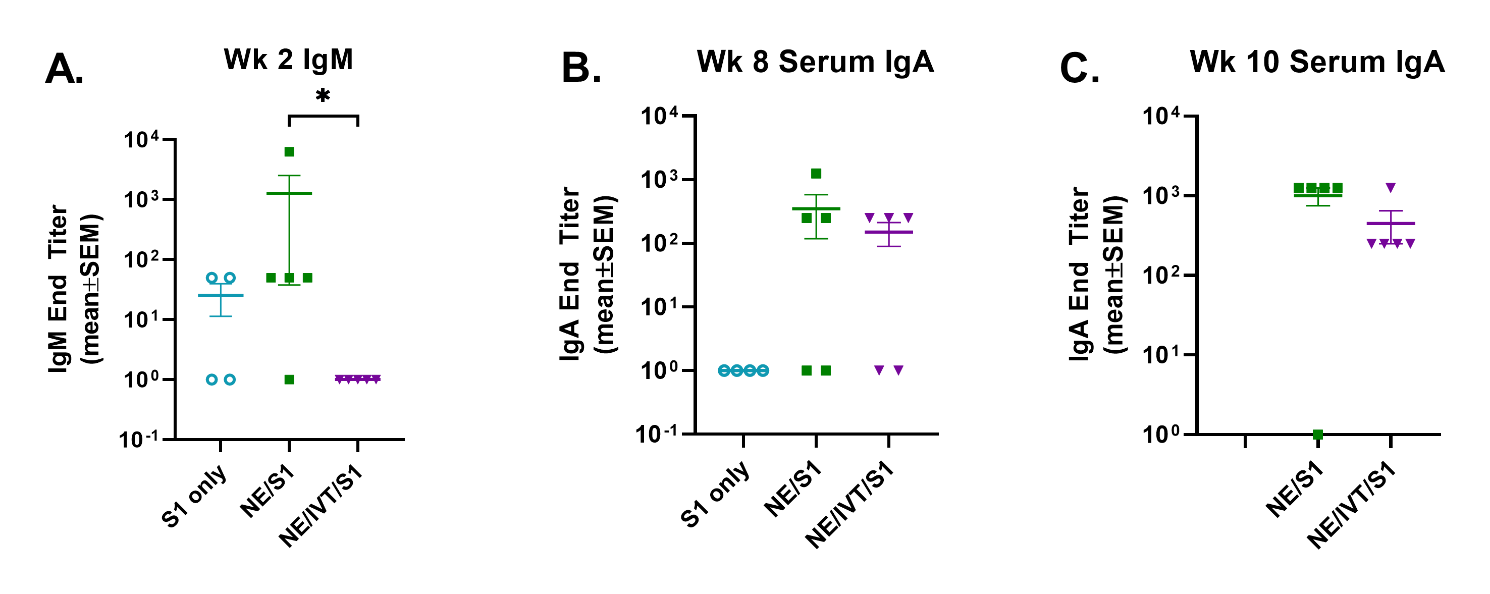


**Supplemental Figure S4.** **Serum RBD-specific IgM, IgG and IgA titers induced by immunization with RBD IN with NE/IVT or IM with Addavax.** Mice were immunized IN with 10 μg RBD alone, or with 20% NE/0.5 μg IVT DI and responses were compared to mice immunized IM with 10 μg RBD with 50% Addavax (Advx). (A) Serum RBD-specific IgM measured 2 weeks after the first immunization. Serum RBD-specific IgG was measured at (B) 8 and (C) 10 wks post-initial immunization (prime/boost, prime/boost/boost, respectively) and (C) serum RBD-specific IgA was measured 10 wks post-initial immunization (prime/boost/boost). (*p<0.05, **p<0.01 by Mann-Whitney U test).


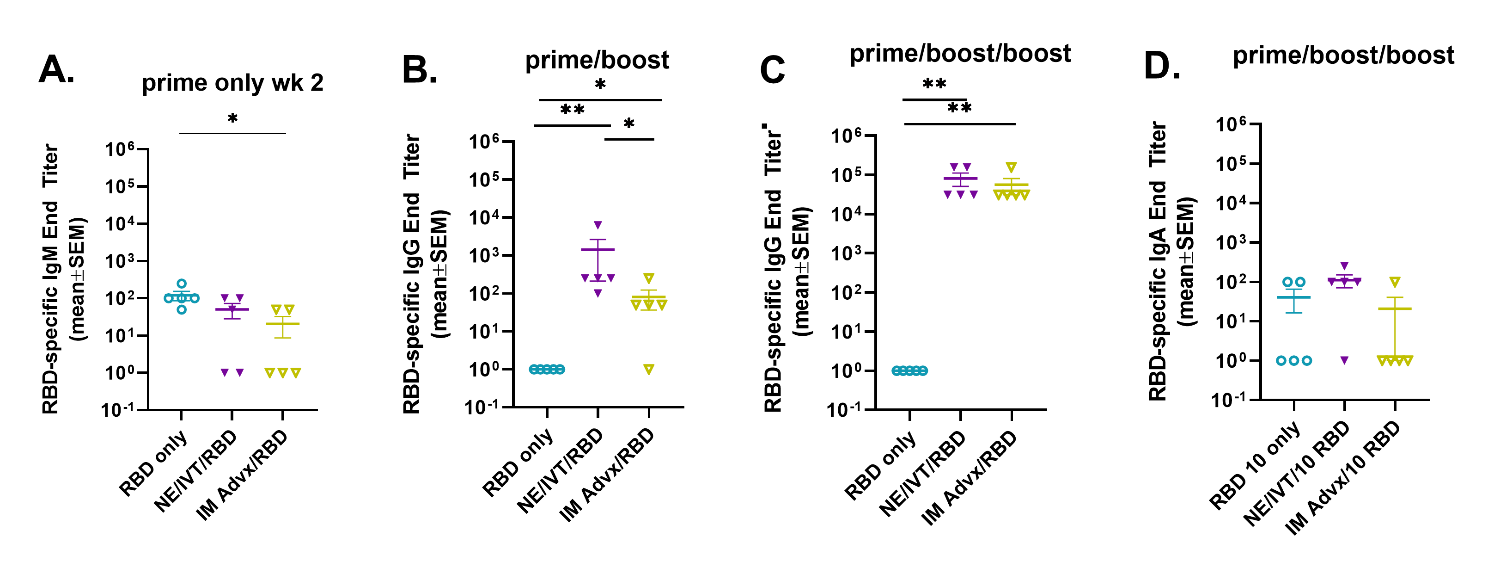

Supplement: Supplementary file 1 [file DataSheet_1.docx]
